# *RET* c.1901G>A and Novel *SLC12A3* Mutations in Familial Pheochromocytomas

**DOI:** 10.3390/genes13050864

**Published:** 2022-05-12

**Authors:** Lin Zhao, Kun-Qi Yang, Peng Fan, Ding-Xu Gong, Lin Zhang, Yi-Ting Lu, Xu Meng, Xian-Liang Zhou

**Affiliations:** 1 Department of Cardiology, Fuwai Hospital, National Center for Cardiovascular Disease, Chinese Academy of Medical Sciences and Peking Union Medical College, No. 167, Beilishi Road, Beijing 100037, China; zhaolin@fuwai.com (L.Z.); zhp123abd@sina.com (K.-Q.Y.); fanpeng126pumc@126.com (P.F.); epusky@126.com (L.Z.); yitinglu@163.com (Y.-T.L.); mengxu1219@hotmail.com (X.M.); 2 Department of Cardiac Surgery, Fuwai Hospital, National Center for Cardiovascular Disease, Chinese Academy of Medical Sciences and Peking Union Medical College, Beijing 100037, China; gongdingxu@fuwai.com

**Keywords:** hypertension, pheochromocytoma, *RET* proto-oncogene, *SLC12A3* gene, MEN2A

## Abstract

Familial PHEOs (pheochromocytomas) are inherited as an autosomal dominant trait, and inherited PHEOs can be one clinical phenotype of clinical syndromes, such as multiple endocrine neoplasia type 2A (MEN2A). In recent years, there has been a lot of controversy about the factors affecting the penetrance of PHEOs in MEN2A, of which the effects of *RET* (rearranged during transfection) proto-oncogene mutations are the primary concern. In this report, we performed genetic screening of patients in one family presenting with PHEOs and found they carried a *RET* c.1901G>A mutation. They were ultimately diagnosed with familial MEN2A. We found that MEN2A patients with the *RET* c.1901G>A mutation tended to have bilateral PHEOs that appeared earlier than medullary thyroid carcinoma. Genetic analysis showed that the patients also carried novel *SLC12A3* (solute carrier family 12 member 3) variants, which are highly associated with Giteman syndrome. The results of protein structure prediction models suggest this *SLC12A3* mutant has altered both the protein structure and the interaction with surrounding amino acids. Further studies of the phenotypes and related mechanisms of the gene mutations are required to guide individual assessment and treatment.

## 1. Introduction

PHEO (pheochromocytoma, OMIM #171300) is a neuroendocrine tumor deriving from the adrenal medulla, which may be either sporadic or familial. It is a rare etiology causing secondary hypertension [1]. Patients with PHEO typically manifest with the classic triad of severe headaches, palpitations, and excessive sweating, which are always episodic [2]. Surgical excision of functional tumors, also known as catecholamine–producing tumors, is proven to be effective in relieving symptoms and improving prognosis.

Familial PHEOs are inherited as an autosomal dominant trait. Neumann and coworkers indicated that more than 35% of PHEOs were attributable to germline disease-causing mutations [3]. The *RET* (rearranged during transfection) proto-oncogene, located on chromosome 10q11.2, was first identified to be associated with inherited PHEOs in 1993 [4]. Since then, at least 20 susceptibility genes have been detected to have germline mutations, such as *NF1* (neurofibromatosis type 1), *TMEM127* (transmembrane protein 127), *FH* (fumarate hydratase), *VHL* (Von Hippel-Lindau), and others [2,3]. Genetic sequencing is now recommended for all PHEO patients. Inherited PHEO can also be one phenotype of specific clinical syndromes, such as multiple endocrine neoplasia type 2A and B (MEN2A, OMIM #171400 and MEN2B, #162300), *NF*1 (OMIM #162200) and *VHL* syndrome (OMIM #193300) [3].

In this study, we performed genetic screening for patients presenting with PHEO and found that they carried a *RET* c.1901G>A mutation. They were ultimately diagnosed with familial MEN2A. In recent years, there has been a lot of controversy about the factors affecting the penetrance of PHEOs in MEN2A. For example, Mucha et al. [5] reported that the penetrance and age at diagnosis of PHEOs in MEN2A were correlated with *RET* mutation position: the penetrance was estimated as 47% in *RET* exon 11 vs. 30% in exon 10 carriers. Castinetti et al. [6] showed that even in *RET* codon 634 mutation carriers, the penetrance could be highly variable depending on the geographical area of the patient’s origin, suggesting the roles of unknown modifiers. In this study, we found the MEN2A family carried a *RET* c.1901G>A mutation, and we aimed to determine the effects of this mutation on the penetrance of PHEO in MEN2A. In addition to the *RET* c.1901G>A mutation, we found that the patients also had a novel variant in *SLC12A3* (solute carrier family 12 member 3), which is highly associated with Gitelman syndrome (GS, OMIM# 263800) [7]. We used the protein structure prediction and comparison models to evaluate the function of the *SLC12A3* mutant. At present, no studies have reported both *RET* and *SLC12A3* mutations in the same patient or family. We are the first to describe compound *RET* c.1901G>A and *SLC12A3* mutations in a family with MEN2A. Our study also underscores the importance of genetic testing for the prevention, treatment, and prognosis of diseases.

## 2. Materials and Methods

### 2.1. Study Patient Description

**Figure 1:**
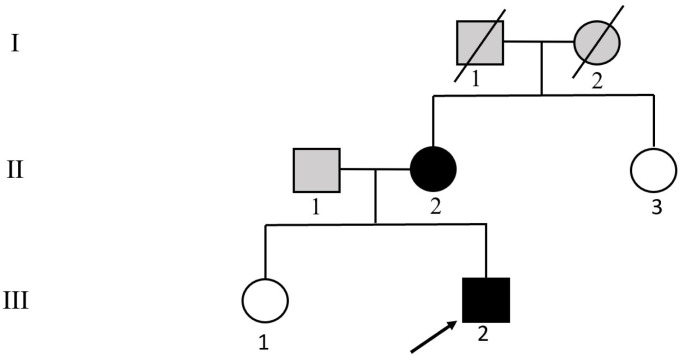
Family pedigree. Black-filled symbols, subjects carrying the identified *RET* c.1901G>A and *SLC12A3* c.3070_3079delinsCAG mutations; empty symbols, subjects without identified mutations; gray-filled symbols, not sequenced; the black arrow indicates the proband.

**Figure 2:**
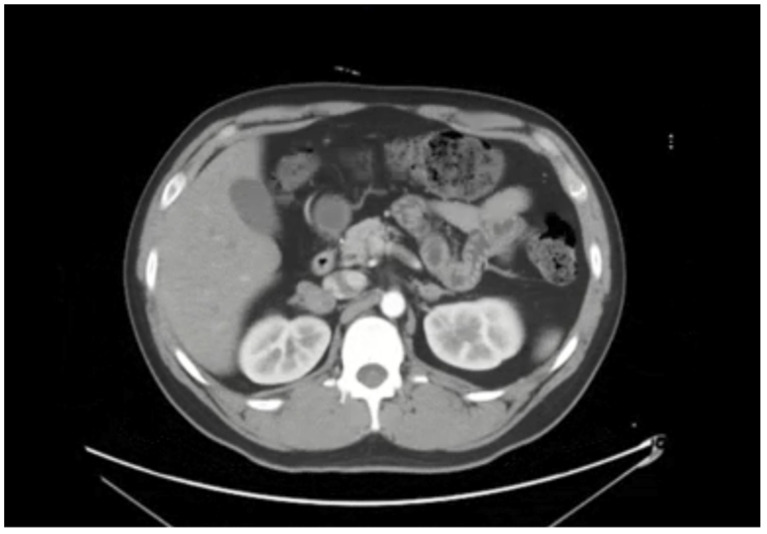
The computed tomography of the proband showed multiple nodules on both sides of the adrenal glands.

The proband (Figure 1, individual III-2), a 28-year-old male with symptoms of paroxysmal palpitation with sweating and headache, was admitted to Fuwai Hospital, Beijing, China. The index patient had suffered from this physical discomfort from the age of 20, but did not receive any further examination and treatment at that time. At the first medical consultation in Fuwai Hospital in August 2020, the patient’s BP (blood pressure) was monitored as 160/103 mmHg during the onset of symptoms, and 140/93 mmHg after onset. Biochemical examination showed that blood and urine catecholamine metabolites were elevated. Computed tomography (CT) of the adrenal glands showed multiple nodules on both sides (Figure 2, the larger tumor was located on the right side with a size of 3.1 cm × 2.8 cm). During the investigation of his family members, we found his mother (Figure 1, individual II-2) was diagnosed with bilateral PHEOs at the age of 34 and had already been treated with surgery. The proband’s grandmother (Figure 1, individual I-2) died at the age of 55 having suffered severe hypertension during her lifetime. The study enrolled most of the family members. The pedigree chart is shown in Figure 1. Peripheral venous blood was collected from each subject for biochemical and genetic analyses. A clinical survey was also conducted for each participant.

### 2.2. Imaging and Laboratory Examinations

Imaging examinations were performed, including echocardiography, abdomen ultrasound, thyroid ultrasound, parathyroid ultrasound, somatostatin receptor scintigraphy, CT of the adrenal glands, kidneys, and renal arteries, iodine–131-metaiodobenzylguanidine (^131^I-MIBG) scintigraphy, and somatostatin receptor scintigraphy. General biochemical measurements associated with hypertension and MEN2A were covered in the laboratory testing, such as serum electrolytes, serum calcitonin, serum catecholamine metabolites, serum thyroid hormone, serum parathormone, urine catecholamine metabolites, urine protein, microalbuminuria, and blood gas analysis.

### 2.3. Molecular Analysis and Bioinformatics Analysis

Whole exome sequencing (WES) was performed for the proband. Genomic DNA (Deoxyribonucleic acid) was extracted from peripheral blood samples from the proband. Qualifying genomic DNA isolated from leukocytes was randomly fragmented, and the sizes of the library fragments were mainly distributed between 180 and 280 bp. We performed DNA end repair of the fragments, and an A base was added at the 3′ end of each strand. Illumina adapters were added to both ends of the library DNA fragments. We used ligation-mediated PCR (polymerase chain reaction) to amplify the size-selected DNA fragments, which were purified and hybridized to the exome array (by SureSelect Target Enrichment System kit, Agilent Technologies, Inc., Santa Clara, California, the United States) for enrichment. Non-hybridized fragments were then washed out, and captured products were circularized. The exon DNA library was then amplified by PCR. Each resulting qualifying captured library was then loaded onto the Illumina PE150, and high-throughput sequencing was performed for each captured library. Through base calling analysis, the raw image data file obtained by the Illumina sequencing platform was converted into raw data sequenced reads and stored in FASTQ format.

Bioinformatics analysis began with the sequencing data. First, data filtering of the raw data produced clean data. All clean data were mapped to the human reference genome (GRCh37/HG19). Then, Burrows–Wheeler Aligner (BWA) software was used to perform the alignment [8]. Local realignment around indels and base quality score recalibration was performed using a Genome Analysis Toolkit (GATK) [9,10]. Duplicate reads were removed using Picard tools. All genomic variations were identified using GATK. Then, all variants were annotated with ANNOVAR [11]. The SIFT, Polyphen1, Polyphen2, and M-CAP [12] software programs were used to predict if variants were deleterious. According to the standards and guidelines for the Interpretation of Sequence Variants, the recommendation of the American College of Medical Genetics and Genomics (ACMG) and the Association for Molecular Pathology in 2015 [13], variants could be classified as pathogenic, likely pathogenic, of uncertain significance, likely benign, and benign.

After the filtering process, Sanger sequencing was used to validate the gene mutations in other members in this family. The primer sequences were as follows: *RET*: forward, 5′-TAAATGGCAGTACCCATGCTC-3′; reverse, 5′-ATCCACGGAGACCTGGTTC-3′, and *SLC12A3*: forward, 5′-ACGGTAAACAGACTCGCTG-TTC-3′; reverse, 5′-GGTGAGATCATCAGATGCTGC-3′. The sequences of the PCR products were determined using the ABI 3730XL Genetic Analyzer (ABI, Foster City, CA, USA).

### 2.4. Protein Structure Prediction

The amino acid sequence of the protein encoded by *SLC12A3* was obtained from Uniprot, and the crystal structure in the protein data bank was searched using NCBI’s Protein BLAST tool [14]. In the search results, the sequence identity between *SLC12A3* and the crystal structure 6NPL [15] indicated that they have high homology. Therefore, 6NPL can be used as a template for the construction of the protein encoded by *SLC12A3* and its mutants. The Modeller v9.19 program [16] was used to perform homology modeling of the protein encoded by *SLC12A3* to obtain a reasonable three-dimensional structure model of the target protein and then to optimize the molecular mechanics. The PROCHECK (version 3.5.4) and Verify 3D [17] programs were used to evaluate the optimized protein model to ensure that the structure of the protein was reasonable.

## 3. Results

### 3.1. Clinical Features

The proband was admitted to the hypertension center for further evaluation. Biochemical measurements (Table 1) showed that he had elevated serum and urine catecholamine metabolites and elevated calcitonin and CEA (carcinoembryonic antigen). Serum thyroid function, serum calcium, serum phosphorus, serum potassium, serum magnesium, and blood gas analysis were measured several times and they were all within the normal range. A normally sized heart was shown by echocardiogram and X-ray. Multiple thyroid nodules were found by ultrasound. Ultrasound did not find obvious structural abnormalities of the parathyroid glands. The somatostatin receptor imaging of the proband showed high expression of the adrenal somatostatin receptor on both sides (left 1.1 cm × 1.2 cm, right 2.5 cm × 3.2 cm). ^131^I-MIBG showed abnormal findings in the right adrenal area. The proband’s mother also had elevated urine catecholamines before surgery (Table 1) and abnormalities were found in the thyroid at this visit. Serum thyroid function, serum calcium, serum phosphorus, serum potassium, serum magnesium, serum phosphorus, and blood gas analysis were also measured within the normal range. The mother had elevated parathormone, while the proband did not. The other relatives were healthy without any symptoms or signs.

### 3.2. Genetic Findings

**Figure 3:**
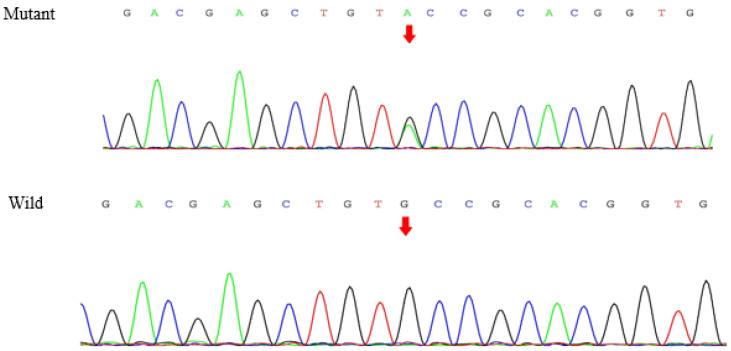
Sanger sequencing identified the mutation in *RET*.

**Figure 4:**
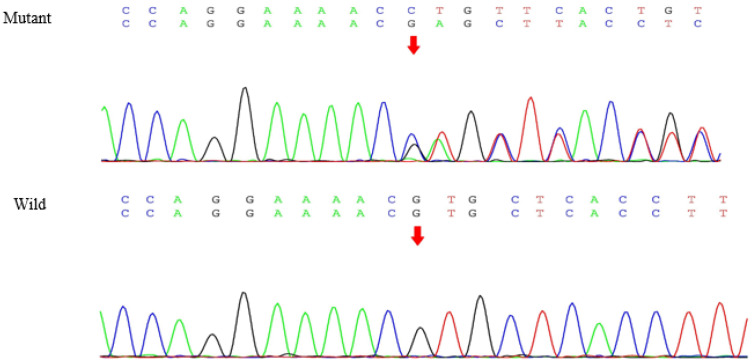
Sanger sequencing identified the mutation in *SLC12A3*.

WES analysis was performed for the proband (Figure 1, individual III-2). After data screening, we detected the c.1901G>A [p.Cys634Tyr, the amino acid changed from cysteine (Cys) to tyrosine (Tyr) in codon 634, C634Y] mutation in exon 11 of *RET* (Figure 3), and a novel variant in exon 26 of *SLC12A3*, c.3070_3079delinsCAG (Figure 4). No other meaningful mutations were identified. The results of WES can be seen in Supplementary Materials. Sanger sequencing found that the mother (Figure 1, individual II-2) carried the same mutations as the proband. The missense mutation in the *RET* gene changes base 1901 from G to A in the coding region, which changes the translated amino acid from cysteine to tyrosine. The frameshift deletion variant of the *SLC12A3* gene involves the deletion of bases 3070 to 3079 in the coding region and the insertion of CAG bases at this position. According to Mutalyzer (https://mutalyzer.nl/, accessed on 20 October 2020), this variant of *SLC12A3* leads to the change in the translated amino acids, that is, beginning from (and including) the 1024 amino acid position and terminating after translation of another ten amino acids (p.Val1024Glnfs*10). Furthermore, the *RET* c.1901G>A mutation is predicted to be deleterious using Provean (http://provean.jcvi.org-/index.php, accessed on 20 October 2020), PolyPhen-2 (http://genetics.bwh.h-arvard.edu/pph2, accessed on 20 October 2020), SIFT (http://sift.jcvi.org, accessed on 20 October 2020), and Mutation Taster (http://www.mutationtaster.org, accessed on 20 October 2020). The *SLC12A3* c.3070_3079delinsCAG mutation is predicted to be disease-causing in Mutation Taster. According to the 2015 ACMG Guidelines [13], the *SLC12A3* c.3070_3079delinsCAG mutation is of uncertain significance.

### 3.3. Protein Structure Prediction of the Novel Variant in SLC12A3

#### 3.3.1. Protein Model of the *SLC12A3* Gene and Its Mutant

**Figure 5:**
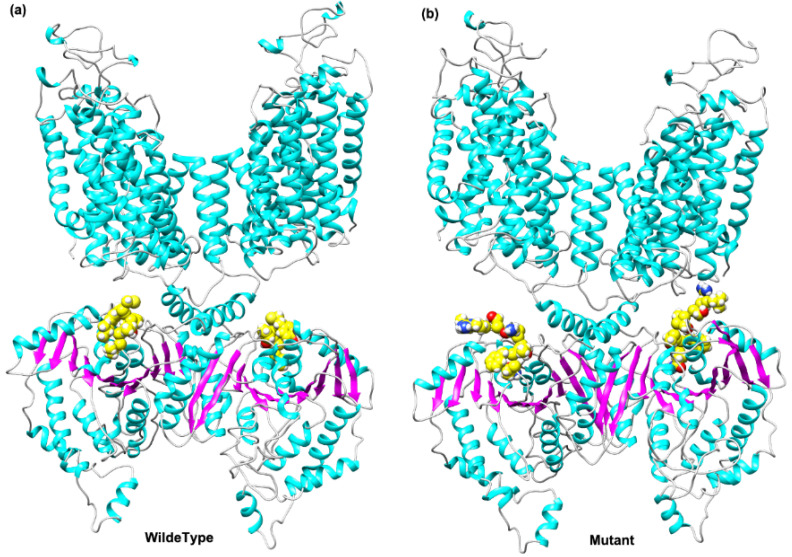
Structural models of the protein encoded by *SLC12A3* (**a**) and the mutant (*SLC12A3* c.3070_3079delinsCAG) (**b**). Yellow spheres indicate mutated amino acid residues.

Figure 5 shows the structure prediction models of the protein encoded by *SLC12A3* and the mutant (*SLC12A3* c.3070_3079delinsCAG). The wild-type protein has a dimer structure composed of two chains, and the mutation site is located in the C-terminal region of the protein. Mutations in this region may result in structural changes in the protein, thereby affecting the function of the protein.

#### 3.3.2. The Binding Mode of the Mutant Amino Acids with the Surrounding Amino Acids

**Figure 6:**
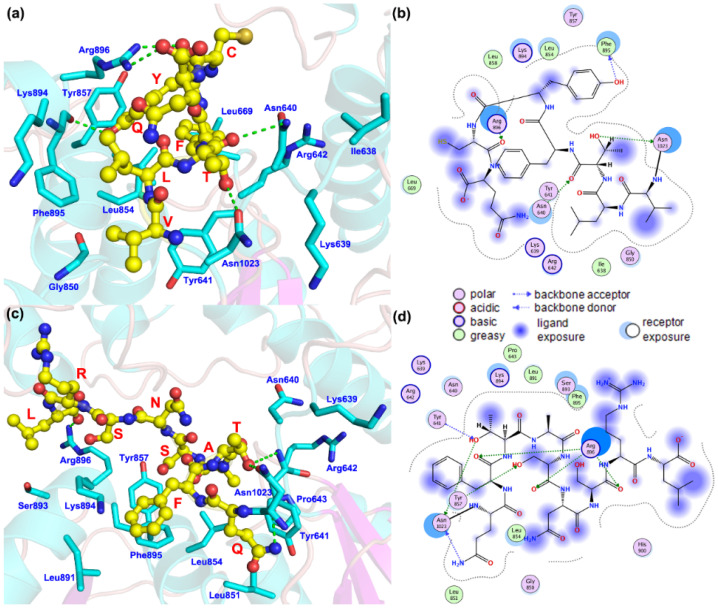
(**a**,**b**): Interaction pattern between the C-terminal amino acids and surrounding amino acid residues in the protein encoded by *SLC12A3*. (**c**,**d**): Interaction pattern between the C-terminal amino acids and the surrounding amino acid residues in the predicted protein encoded by *SLC12A3* c.3070_3079-delinsCAG. Yellow spheres indicate mutated amino acid residues.

To study the effects of *SLC12A3* c.3070_3079delinsCAG, Figure 6 shows the structure of the protein and the location of the mutant. The wild-type C-terminal amino acids are Asn1023-VLTFYC-Gln1030 (asparagine 1023–valine–leucine–threonine–phenylalanine–tyrosine–cysteine–glutamine 1030), and for the mutant, the C-terminal amino acids of the predicted protein structure are Asn1023-QFTASNSR-Leu1032 (Asn1023–glutamine–phenylalanine–threonine–alanine–serine–asparagine–serine–arginine–leucine 1032). The interaction pattern between the terminal amino acids and the amino acid residues of the protein changed significantly. In the wildtype protein, this sequence can form hydrogen bond interactions with Asn640, phenylalanine 895 (Phe895), arginine 896 (Arg896), and Asn1023 and can form strong hydrophobic interactions with isoleucine 638 (Ile638), Leu669, Leu854, Leu858, and Phe895. In the predicted structure of the mutant, the number of amino acids increased and mainly formed hydrogen bonds with tyrosine 857 (Tyr857), Arg896 and Asn1023, and hydrophobic interactions with proline 643 (Pro643), Leu851, Leu854, Leu891, and Phe895.

In summary, this novel variant of *SLC12A3* can not only destroy the hydrogen bonds between the amino acids of the protein and nearby amino acids but also greatly affects other interactions, including hydrophobic and polar interactions. By analyzing the location of the mutation points of the protein and the structure of the mutant, it is theoretically explained that *SLC12A3* c.3070_3079delinsCAG may affect the interactions of amino acid residues, destroy the stability of the protein itself, and ultimately affect the function of the protein.

### 3.4. Patient Follow-Up

The proband underwent surgery of the right adrenal tumor, which was confirmed to be PHEO by pathology. The proband’s symptoms improved after surgery. Three months after surgery, the 24-h urine catecholamines of the proband were lower than before surgery. Blood gas analysis, serum, and urine electrolytes were all within the normal range. The re-examination of the abdominal and pelvic CT revealed that the right adrenal gland showed postoperative changes, and the size of the left adrenal nodule was basically the same as before. We suggested that the proband had regular evaluation of the left adrenal tumor and catecholamines. If the left adrenal tumor were to enlarge or the serum or urine catecholamines continue to increase, we recommend that the proband should undergo resection of the left adrenal tumor. According to the 2015 American Thyroid Association Guidelines for the Management of Medullary Thyroid Carcinoma [18], patients with *RET* codon 634 mutations are at high risk. The proband has high calcitonin and CEA levels, suggesting that he may have MTC (medullary thyroid carcinoma). We recommend that the serum calcitonin and CEA levels of the proband be monitored, and thyroidectomy should be considered. There is currently no evidence that the proband had HPT (hyperparathyroidism). The proband’s mother was discovered to have abnormalities in the thyroid and was diagnosed with MTC after thyroidectomy. She was also diagnosed with HPT, but she suffered from no relevant clinical symptoms.

## 4. Discussion

In our study, the proband’s mother was diagnosed with bilateral PHEOs, MTC, and HPT, and in combination with the *RET* c.1901G>A (C634Y) mutation, she was diagnosed with MEN2A. The proband was diagnosed with right PHEO. The *RET* C634Y mutation and the high calcitonin and CEA levels of the proband suggest that he may have MTC. Although there is no evidence that he suffers from HPT, since the proband and his mother have the same genetic mutations, the proband’s disease progression may be similar to that of his mother. Since the proband’s grandmother also had similar symptoms during her lifetime, we speculate that the genetic mutations were inherited from the grandmother. An interesting phenomenon was that the proband’s mother presented with PHEO-related symptoms first and was diagnosed with MTC approximately 20 years later. The proband presented PHEO-related symptoms eight years ago and denied a history of thyroid abnormalities, but possible MTC was found at this presentation. Our findings suggest that MEN2A patients with the *RET* C634Y mutation tended to have bilateral PHEOs and may be more prone to PHEO before MTC.

### 4.1. The RET Mutation and Familial PHEOs in MEN2A

The human *RET* proto-oncogene maps to 10q11.2 and encodes a transmembrane receptor tyrosine kinase for members of the glial cell line-derived neurotrophic factor family and associated ligands [19,20]. Germline mutations in *RET* cause MEN2. MEN2A is the most common manifestation of MEN2 and is characterized by MTC and PHEO plus primary HPT [20]. To date, complete curation of close to 200 *RET* germline variants has been reported and is hosted in the continually-updated ARUP MEN2 database [21,22]. Mutations in only a few codons in the *RET* proto-oncogene, mainly located in exons 10, 11, 13, 14, and 16, predispose individuals to MEN2 [23,24,25]. In the Chinese population, the most frequent *RET* mutation is localized at codon 634 of exon 11, and the C634Y mutation is the most common, followed by C634R (the amino acid changes from Cys to Arg in codon 634) [26].

With the discovery that mutations in *RET* cause the MEN2 syndromes and the recognition that there is a correlation between genotype and phenotype, the focus on early diagnosis shifted to direct DNA analysis. However, it soon became apparent that there was great heterogeneity in the age of onset and penetrance of PHEO, which may be related to the different mutations in *RET*. For example, *RET* codon 634 mutations are associated with a high penetrance of PHEO, which in one study increased with age, being 25% by age 30 years, 52% by age 50 years, and 88% by age 77 years [27]. In Castinetti et al.’s study [28], the germline *RET* mutations of MEN2 were all of the missense type and were mainly located in exons 10 (9%), 11 (84%), and 16 (5%), and *RET* codon 634 mutations were the most common mutations. Age at diagnosis was the characteristic that differed between patients with different *RET* mutations: the mean age at diagnosis of first PHEO was 45.4 years for exon 10, 37.2 years for exon 11, and 26.7 years for exon 16 (*p* = 0.048). In another study [29], the mean age of developing the first PHEO with the C634Y mutation was 36.0 ± 11.3 years, which is consistent with the results in this study. Mucha et al. [5] explained this in more detail that the highest penetrance of PHEO was patients with Cys634Arg substitution, followed by Cys634Tyr, Cys634Trp (the amino acid changed from Cys to tryptophan in codon 634), and Cys634Gly (the amino acid changed from Cys to glycine in codon 634). Individuals carrying two *RET* polymorphic alleles were younger at diagnosis when compared with those with one or no polymorphisms (*p* = 0.006) [30].

For bilateral PHEOs in MEN2A, another study [29] showed that, at the age of 40 years, for patients with the C634R mutation, the estimated penetrance of bilateral PHEOs was 59.3% and for those with C634Y, this was 25.2%. However, by the age of 80 years, for patients with the C634R or C634Y mutations, the estimated risk of bilateral PHEOs approached each other (78.6% vs. 83.2%). In Castinetti et al.’s study [28], of 563 patients with PHEOs, 44% had bilateral and 56% unilateral PHEOs at first presentation. Of 313 patients with unilateral PHEOs, 30% subsequently developed a contralateral PHEO at a mean of nine years later. Genetic predisposition to tumors causes specific gene-informed recommendations for the treatment of tumors and includes considerations for the prevention of second tumors in the same organs or potential tumor relapses. However, as shown above, in genetic PHEO, bilateral PHEOs are frequent, and it needs to be noted that there may be a long interval until contralateral tumors are discovered. Therefore, lifelong monitoring for PHEO development is necessary. The mother of the proband had already been diagnosed with bilateral PHEOs at the first visit and underwent surgery with no recurrence or metastasis. The proband was only determined to have right PHEO at this time, and he needs to be closely followed up.

The factors influencing the sequence of PHEO and MTC in MEN2A are still unclear. This may also be related to *RET* mutation. Mutations in codon 634 have a higher potency for cell neoplastic transformation than those in codons 609 and 611; this finding may result from modulations in the expression of the mature *RET*-encoded protein receptors in the cell membrane [31,32]. Therefore, most patients with *RET* 634 mutations will have adrenal and parathyroid tumors in addition to thyroid tumors. In our study, with the *RET* C634Y mutation, the proband’s mother not only had bilateral PHEOs but also had MTC and HPT; the proband was diagnosed with right PHEO and probable MTC, which supports the above results. Castinetti et al.’s study [28] showed that PHEO was detected in 47% of MEN2 patients; 54% developed symptomatic PHEO after a prior diagnosis of MTC; in 30%, PHEO was detected at the same time as MTC; and in 16%, PHEO was diagnosed before MTC. However, this study did not specify whether the sequence of PHEO and MTC was related to different *RET* mutations. In our study, the proband’s mother was first diagnosed with bilateral PHEOs and was then diagnosed with MTC about 20 years later. The proband presented with PHEO-related symptoms eight years ago and denied a history of thyroid abnormalities, but possible MTC was found at this presentation. Our findings may suggest that MEN2A patients with the *RET* C634Y mutation may be more prone to PHEO before MTC. This conclusion may provide more individualized guidance for disease prevention in MEN2A patients and also has the potential to inform more accurate timing of surgery to induce the predictable side effects of thyroidectomy [18]. However, due to the small number of cases, the generalization of this conclusion needs to be very cautious. Further research is required to explore this issue.

### 4.2. Consideration of the Novel SLC12A3 Mutation

The human *SLC12A3* gene is located on 16q13 and includes 26 exons, encoding a thiazide-sensitive sodium-chloride cotransporter (NCCT) [33]. Mutations in *SLC12A3* are thought to be related to GS, which is inherited in an autosomal recessive pattern [7]. The effects of decreased NCCT activity will be similar to persistent thiazide diuretic effects. Therefore, GS is usually characterized by metabolic alkalosis, hypokalemia, and hypomagnesemia of renal origin, as well as hypocalciuria [7]. The prevalence of GS is approximately 1–10 per 40,000 people, and potentially higher in Asia [7]. So far, more than 400 *SLC12A3* mutations have been found to be related to GS in the human gene mutation database, in which compound heterozygous mutations excel homozygous mutations, and no hotspot mutations are found [34]. The novel variant of *SLC12A3* described in our study was not reported before. In this study, the proband and his mother who carry this novel mutation do not have the typical clinical manifestations and biochemical abnormalities of GS. Although some patients who meet the diagnostic criteria of GS are heterozygotes, in a large cohort of such patients, a considerable number of patients have many genome rearrangements on the other allele [35]. The patients in our study do not fit this situation. Therefore, the absence of a clinical manifestation of GS in the patients in this family may be a result of heterozygosity.

### 4.3. The Connection between SLC12A3 Mutations and MEN2A

The *SLC12A3* gene encodes the renal NCCT [33], and the human NCCT is specifically expressed in the distal convoluted tubule [33,36]. We also searched the distribution of organs expressing the *SLC12A3* gene in the human body on the human protein atlas (http://www.protein-atlas.org/, accessed on 31 October 2021). The result showed that the *SLC12A3* gene is mainly expressed in the kidney and testis, not in the adrenal or thyroid gland or parathyroid glands, which are the main target organs of MEN2A. This phenomenon explains why the *SLC12A3* gene variants may not be significantly related to the MEN2A syndrome phenotype in our study. According to the 2015 ACMG Guidelines [13], *SLC12A3* c.3070_3079delins-CAG is classified as of uncertain significance, while it is predicted to be disease-causing in Mutation Taster. Furthermore, by protein structure prediction, it is theoretically explained that the novel variants of *SLC12A3* we found in this family affect the interaction of amino acid residues, destroy the stability of the protein itself, and ultimately affect the function of the protein. Based on this, we suggest that patients with this novel variant are followed up regularly to help clarify the significance of the mutations.

Our study has several limitations. First, the sample size was small. However, this is related to the rare incidence of the disease and the novel gene mutations we first discovered. In addition, the possibility of reporting bias with overestimation of uncharacteristic presentations cannot be ruled out. However, this is again unavoidable due to the infrequent occurrence of this disease. Finally, these results should be generalized with caution owing to the bias.

## 5. Conclusions

In summary, we are the first to describe compound *RET* c.1901G>A and the novel *SLC12A3* c.3070_3079-delinsCAG mutation in a family with MEN2A. MEN2A patients with the *RET* c.1901G>A mutation tend to have bilateral PHEOs and their PHEOs may appear earlier than MTC. The novel variant of *SLC12A3* is not related to the phenotype of MEN2A, but affects the function of the protein. Preventative medicine and cancer genetics are highly interlinked specialties of modern medicine that are increasing in importance. More studies of the phenotypes and related mechanisms of the gene mutations are needed to guide individual assessment and treatment.

## Figures and Tables

**Table 1:** Laboratory examinations compared between the proband and his mother before surgery.

	Normal Reference Value	Proband	Proband’s Mother
NE	<0.548 ng/mL	1.301 ng/mL	—
E	<0.2 ng ng/mL	1.907 ng/mL	—
NMN	<0.9 nmol/L	1.7 nmol/L	—
MN	<0.5 nmol/L	2.49 nmol/L	—
Dopamine	<0.08 ng ng/mL	<0.02 ng/mL	—
CEA	<5 ng/mL	7.3 ng/mL	14.29 ng/mL
Calcitonin	<10 pg/mL	44.56 pg/mL	—
Parathormone	15–65 pg/mL	55.02 pg/mL	169.7 pg/mL
serum calcium	2.09–2.54 mmol/L	2.3 mmol/L	2.50 mmol/L
serum phosphorus	0.89–1.6 mmol/L	1.32 mmol/L	1.29 mmol/L
serum potassium	3.5–5.5 mmol/L	4.2 mmol/L	4.53 mmol/L
serum magnesium	0.6–1.4 mmol/L	1.0 mmol/L	0.92 mmol/L
24hU-NE	16.49–40.65 µg/24 h	46.33 µg/24 h	—
24hU-E	1.74–6.42 µg/24 h	18.08 µg/24 h	—
U-Dopamine	120.93–330.59 µg/24 h	257.06 µg/24 h	—
U-catecholamine	59.1–266 nmol/24 h	—	784.6 nmol/24 h
U-VMA	8.6–76.3 umol/24 h	—	89.1 umol/24 h

Abbreviations: NE: norepinephrine; E: epinephrine; NMN: normetanephrine; MN: metanephrine; CEA: carcinoembryonic antigen; U: urine; 24hU-NE: 24 h-urine norepinephrine; 24hU-E: 24 h urine epinephrine; U-VMA: urine vanillymandelic acid.

## Data Availability

Data are contained within the article and Supplementary Materials.

## Supplementary Materials

The following supporting information can be downloaded at: https://www.mdpi.com/article/10.3390/genes13050864/s1. Supplementary Material S1. The information of annotated gene mutations 1; Supplementary Material S2. The information of annotated gene mutations 2.
